# Characterization of Differentially Expressed Genes Involved in Pathways Associated with Gastric Cancer

**DOI:** 10.1371/journal.pone.0125013

**Published:** 2015-04-30

**Authors:** Hao Li, Beiqin Yu, Jianfang Li, Liping Su, Min Yan, Jun Zhang, Chen Li, Zhenggang Zhu, Bingya Liu

**Affiliations:** 1 Shanghai Key Laboratory of Gastric Neoplasms, Shanghai Institute of Digestive Surgery, Ruijin Hospital, Shanghai Jiao Tong University School of Medicine, Shanghai 200025, People’s Republic of China; 2 Department of Oncology, Ruijin Hospital, Shanghai Jiao Tong University School of Medicine, Shanghai 200025, People’s Republic of China; 3 Department of Surgery, Ruijin Hospital, Shanghai Jiao Tong University School of Medicine, Shanghai 200025, People’s Republic of China; University of Jaén, SPAIN

## Abstract

To explore the patterns of gene expression in gastric cancer, a total of 26 paired gastric cancer and noncancerous tissues from patients were enrolled for gene expression microarray analyses. Limma methods were applied to analyze the data, and genes were considered to be significantly differentially expressed if the False Discovery Rate (FDR) value was < 0.01, *P*-value was <0.01 and the fold change (FC) was >2. Subsequently, Gene Ontology (GO) categories were used to analyze the main functions of the differentially expressed genes. According to the Kyoto Encyclopedia of Genes and Genomes (KEGG) database, we found pathways significantly associated with the differential genes. Gene-Act network and co-expression network were built respectively based on the relationships among the genes, proteins and compounds in the database. 2371 mRNAs and 350 lncRNAs considered as significantly differentially expressed genes were selected for the further analysis. The GO categories, pathway analyses and the Gene-Act network showed a consistent result that up-regulated genes were responsible for tumorigenesis, migration, angiogenesis and microenvironment formation, while down-regulated genes were involved in metabolism. These results of this study provide some novel findings on coding RNAs, lncRNAs, pathways and the co-expression network in gastric cancer which will be useful to guide further investigation and target therapy for this disease.

## Introduction

Gastric cancer (GC) is one of the most common cancers worldwide, and its incidence is particularly high in Eastern Asia, especially in China. Approximately 952,000 new cases of stomach cancer were diagnosed worldwide in 2012, and half of them occurred in Eastern Asia (mainly in China) [1]. In China, the majority of patients with GC are diagnosed at a late stage with poor prognosis. Therefore, elucidating the molecular mechanisms underlying GC progression is essential to identifying key biomarkers and developing effective targeted therapies.

Over the last decade, gene expression microarrays have become a common tool for examining gene transcript levels in cancer research. Microarray data is used for a wide variety of analyses, such as unsupervised clustering, classification, differential expression analysis, and expression mapping of quantitative trait loci [2]. It not only helps to identify key dysfunctional genes in cancer but provides genome-wide information on gene expression at one time as well[3,4]. In this study, we performed a genome-wide survey of the expression of lncRNAs and mRNAs from paired samples of primary gastric cancer tissues and noncancerous tissues, to profile the differentially expressed lncRNAs and coding RNAs. Study of these data will provide valuable information on the mechanism of carcinogenesis and allow discovery of key genes that may act as future targets of anti-cancer therapy.

## Methods and Materials

### Ethical statement

Written informed consent was obtained from all participants. The study was approved by the Human Research Ethics Committee of Ruijin Hospital, Shanghai Jiao Tong University, School of Medicine.

### Tissue samples

Tissues were taken from primary gastric carcinomas from untreated patients who underwent D2 radical gastrectomy in Shanghai Ruijin Hospital. For each cancer tissue, a paired noncancerous tissue sample was collected from the adjacent region at the same time. The size of each sample was around 0.1cm^3^. All the samples were placed in RNALater within 15 minutes after excision and stored in liquid nitrogen until RNA extraction. In this study, 32 paired tissues were collected for the microarray and 26 paired samples were enrolled for the next-step analysis of GO, pathway and network after quality control using 3D Principal component analysis (3D-PCA) and Cluster analysis.

### Microarray experiments

Agilent SurePrint G3 Human GE 8x60K Microarray (Design ID: 028004) was employed in this study. Total RNA was isolated and amplified using a Low Input Quick Amp Labeling Kit, One-Color (Cat#5190–2305, Agilent technologies, US). Then, the labeled cRNAs were purified by a RNeasy mini kit (Cat#74106, QIAGEN, Germany).

Based on the manufacturer’s instructions, each slide was hybridized with 600ng Cy3-labeled cRNA using a Gene Expression Hybridization Kit (Cat#5188–5242, Agilent technologies, US) and washed by the Gene Expression Wash Buffer Kit (Cat#5188–5327, Agilent technologies, US).

An Agilent Microarray Scanner (Cat#G2565CA, Agilent technologies, US) and Feature Extraction software 10.7 (Agilent technologies, US) were applied to scan each slide with the same settings shown as follow, Dye channel: Green, Scan resolution = 3μm, 20bit. The raw data were normalized by the Quantile algorithm, Gene Spring Software 11.0 (Agilent technologies, US) (detailed in S5 Table).

### Limma

Linear models and empirical Bayes methods were applied to analyze the data in this study. The resulting *P*-values were adjusted using the BH FDR algorithm. There were three standards for us to consider that a gene was significantly differentially expressed, the FDR value was <0.01, *P*-value was <0.01 and the fold change was >2. (detailed in S5 Table)

### GO category

We performed Gene Ontology (GO) analyses to analyze the functions of the differentially expressed genes in our microarray according to the key functional classification of The National Center for Biotechnology Information (NCBI). Generally, Fisher’s exact test and the *χ* ^2^ test were applied to classify the GO category, and the false discovery rate (FDR, $FDR\;=\;1-\frac{Nk}{T}$) was calculated to correct the *P*-value (*N*_*k*_ refers to the number of Fisher’s test *P*-values less than the *χ* ^2^ test *P*-values). The enrichment Re was given by: Re = (*n*_*f*_/*n*)/(*N*_*f*_/*N*) in the significant categories (*N*_*f*_ is the number of differential genes within the particular category, *n* is the total number of genes within the same category, *n*_*f*_ is the number of differential genes in the entire microarray, and *N* is the total number of genes in the microarray.)(detailed in S5 Table).

### Pathway analyses

Pathway annotations of the differential exressed genes were obtained from KEGG (http://www.genome.jp/kegg/). Pathway categories with a FDR <0.01 were marked. The enrichment of significant pathways was given by: enrichment = $\left(\frac{ng}{na}\right)$/$\left(\frac{Ng}{Na}\right)$, which helped us to locate more significant pathways in our study (*n*_*g*_ is the number of differential genes within the particular pathway, *n*_*a*_ is the total number of genes within the same pathway, *N*_*g*_ is the number of differential genes which have at least one pathway annotation, and *N*_*a*_ is the number of genes which have at least one pathway annotation in the entire microarray.) (detailed in S5 Table).

### Gene-Act network

According to the KEGG database, one gene may be involved in several pathways or interact with several other genes. All the gene—gene interactions were pooled together to build the Gene-Act network based on the differential pathways, which helped us to reveal the signaling pathways and key regulatory genes in GC.

### Co-expression network

Gene co-expression Network was built according to the normalized signal intensity of specific expression genes. Degree centrality is defined as the number of links one node has to another, which determines the relative importance of genes. What’s more, k-cores were applied as a method of simplifying the graph topology analyses. Core regulatory factors (genes) which have the highest degrees connect most adjacent genes and build the structure of the network (detailed in S5 Table).

### Real-time quantitative PCR

Total RNA was extracted from tissues using the Trizol reagent (Invitrogen) according to the manufacturer’s instructions. The quantitative real-time polymerase chain reaction (PCR) was performed by using SYBR-green PCR Master Mix in a Fast Real-time PCR 7500 System (Applied Biosystems). The primers of the 10 genes were showed in S4 Table. PCR reactions were performed at 50°C for 2 min, followed by 40 cycles of 95°C for 15 s and 60°C for 1 min. ΔCt was calculated by subtracting the Ct of β-actin RNA (control) from the Ct of the RNA of sample, respectively. ΔΔCt was then calculated by subtracting the ΔCt of the control from the ΔCt of the sample. Fold change was calculated by the equation 2-ΔΔCt.

### Statistical analysis

SPSS software 19 and Microsoft Excel 2010 was used to analyze the data. Expression levels between cancer tissues and adjacent noncancerous tissues were analyzed by paired-sample t-tests. *P*-values below 0.05 were regarded as statistically significant.

## Results

### Microarray analyses

**Fig 1:**
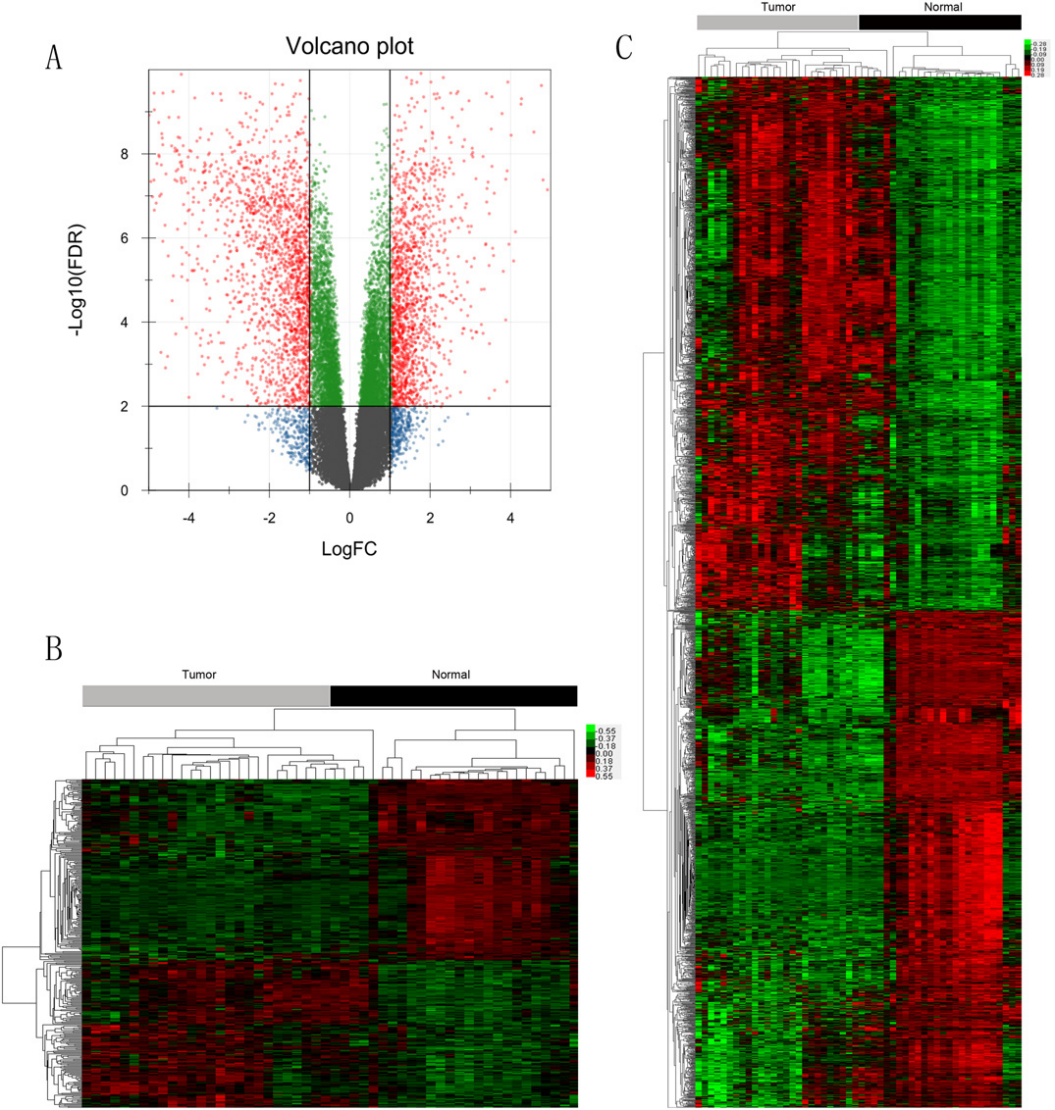
Differentially expressed genes in a gene expression microarray of 26 pairs of gastric cancer and noncancerous tissues. **A)** Volcano plot showing the differential genes (red dots) in the expression microarray (*P*-value <0.01, FDR <0.01). **B)** Clustering heatmap showing the differential lncRNAs. Each column represents one sample and each row represents one differential lncRNA. **C)** Clustering heatmap showing the differential mRNAs. Each column represents one sample and each row represents one differential mRNA.

In total, 42,405 human genes were profiled in our study by using an Agilent G3 Human GE 8x60K microarray. We have submitted our dataset in the repository of “Gene Expression Omnibus” and the accession number was “GSE65801” (http://www.ncbi.nlm.nih.gov/geo/query/acc.cgi?acc=GSE65801). We used linear models and empirical Bayes methods to analyze the data (see Methods). There were 2371 mRNAs and 350 lncRNAs considered as the differentially expressed genes by limma for the next-step analysis (Fig 1A).

Among all 2371 differential mRNAs, there are 1142 mRNAs down-regulated and 1229 mRNAs up-regulated in our observation on alterations of gene expression between gastric cancer and control tissues (Fig 1C). Most of the differential mRNAs have been proven to be correlated with carcinogenesis and metastasis in most types of cancer (Table 1). The genes such as GKN2, PGC, MUC6, CHIA, PSCA and FBP2 were among the top 20 down-regulated genes, while KLK8, SFRP4, INHBA, CLDN1, CST1, FAP, SPP1, OLFM4, and KRT17 were among the top 20 up-regulated genes (Table 1). However, some genes such as HOXC9, FNDC1, STRA6, KCNE2, PGA3 and KCNJ16 haven’t been reported in gastric cancer and their roles remain unknown (Table 1).

**Table 1:** top 40 differential expressed mRNAs in gastric cancer

Gene ID	Gene Symbol	Log2FC	P-value	FDR	Gene ID	Gene Symbol	Log2FC	P-value	FDR
8513	**LIPF**	-11.8913	2.90E-15	1.25E-11	3225	**HOXC9**	5.28242	4.82E-13	3.03E-10
495	**ATP4A**	-10.9406	1.20E-14	3.02E-11	11202	**KLK8[20]**	4.879913	1.28E-09	6.56E-08
200504	**GKN2[5,6]**	-10.7873	1.58E-15	7.96E-12	84624	**FNDC1**	4.769853	6.11E-10	3.82E-08
496	**ATP4B**	-10.3587	1.37E-12	5.14E-10	6424	**SFRP4[21]**	4.727838	3.33E-13	2.18E-10
56287	**GKN1[7,8]**	-10.3119	1.22E-15	7.35E-12	3624	**INHBA[22,23]**	4.543485	4.35E-14	6.15E-11
643834	**PGA3**	-10.0035	1.34E-14	3.11E-11	57214	**KIAA1199**	4.23391	1.12E-12	4.49E-10
9992	**KCNE2**	-8.71399	2.95E-17	2.97E-13	7058	**THBS2[24]**	4.221663	1.76E-12	6.04E-10
5225	**PGC[9,10]**	-8.58129	5.84E-12	1.42E-09	5653	**KLK6[25,26]**	4.083789	2.59E-08	6.53E-07
202915	**TMEM184A**	-8.57672	4.17E-12	1.12E-09	7060	**THBS4**	4.039727	1.92E-07	3.23E-06
5225	**PGC**	-8.45711	2.67E-12	8.22E-10	9076	**CLDN1[27,28]**	3.97693	1.84E-11	3.31E-09
4588	**MUC6[11,12]**	-7.51207	3.62E-09	1.42E-07	1469	**CST1[29]**	3.922162	6.07E-08	1.29E-06
27159	**CHIA[13,14]**	-7.35748	8.68E-11	1.03E-08	2191	**FAP[30]**	3.894022	2.39E-13	1.80E-10
887	**CCKBR[15,16]**	-7.21386	1.42E-17	2.14E-13	6696	**SPP1[31]**	3.877552	3.00E-10	2.33E-08
768239	**PSAPL1**	-6.96121	3.78E-10	2.72E-08	10562	**OLFM4[32]**	3.872285	5.92E-04	0.002384
1113	**CHGA**	-6.79298	1.52E-10	1.50E-08	3872	**KRT17[33]**	3.843196	5.78E-10	3.68E-08
8000	**PSCA[17,18]**	-6.75729	3.69E-09	1.45E-07	1365	**CLDN3[34]**	3.840947	1.01E-05	8.14E-05
3773	**KCNJ16**	-6.61293	3.07E-11	4.80E-09	3219	**HOXB9[35]**	3.77977	3.91E-07	5.68E-06
284340	**CXCL17**	-6.53907	1.54E-07	2.69E-06	7058	**THBS2**	3.750796	3.57E-12	1.01E-09
8789	**FBP2[19]**	-6.36805	1.48E-12	5.45E-10	140453	**MUC17**	3.565985	0.00104394	0.003824
6750	**SST**	-6.3097	9.27E-11	1.07E-08	64220	**STRA6**	3.554919	1.63E-09	7.93E-08

Log2FC<0: down-regulated (left panel), Log2FC>0: up-regulated (right panel)

In addition, we found 193 down-regulated lncRNAs and 156 up-regulated lncRNAs among a total of 350 differential lncRNAs based on the profiling (Fig 1B). Most of the lncRNAs have not been given an official names and their functions remain unknown. However, some have been reported playing critical roles in cancer, such as H19, GUCY1B2, MEG3 and AKR7L (Table 2).

**Table 2:** top 60 differential expressed lncRNAs in gastric cancer

Probe_Set_ID	Name	Gene Symbol	Log2FC	P-value	FDR	Probe_Set_ID	Name	Gene Symbol	Log2FC	P-value	FDR
A_33_P3325763		LRRC3DN	-6.14769	1.5259E-12	5.47E-10	A_24_P52697	**H19[36,37]**	H19	3.516027	8.1109E-08	1.61E-06
A_32_P54274	**DRD5**	DRD5P2	-6.05698	5.9088E-13	3.3E-10	A_19_P00323082		H19	3.339731	6.3201E-08	1.33E-06
A_19_P00317793		LOC388796	-5.65511	2.8645E-10	2.26E-08	A_23_P14255	**GUCY1B2[38]**	GUCY1B2	3.059881	1.603E-07	2.78E-06
A_33_P3261463		FLJ42875	-5.3012	4.0515E-11	5.87E-09	A_19_P00315647		LOC100507165	2.979945	2.0656E-05	0.000146
A_32_P23125	**C20orf56**	LINC00261	-5.28155	7.5952E-07	9.77E-06	A_33_P3266739	**FLJ41200**	FLJ41200	2.729108	1.9325E-06	2.12E-05
A_33_P3285271	**FMO6P**	FMO6P	-4.68528	5.9473E-13	3.3E-10	A_24_P892472	**EMX2OS**	EMX2OS	2.477316	8.0746E-05	0.00045
A_23_P61042		IGH/IGHA2	-4.42815	5.3237E-07	7.3E-06	A_23_P102681		MGC4294	2.31247	1.4528E-08	4.08E-07
A_33_P3357332		C9orf147	-4.17305	3.7913E-09	1.47E-07	A_19_P00322310		LOC100505702	2.208116	1.74E-09	8.36E-08
A_33_P3322874	**LOC100128131**	LOC100128131	-4.15608	5.36E-10	3.47E-08	A_23_P91636	**POM121L9P**	POM121L9P	2.204248	1.3387E-08	3.81E-07
A_33_P3331314		TRA/TRAV8-1	-4.12016	6.9444E-10	4.14E-08	A_19_P00807828		MIR100HG	2.191154	1.5729E-05	0.000117
A_19_P00320047		LOC440993	-4.06041	9.4911E-11	1.09E-08	A_33_P3237850		FLJ39632	2.188395	9.9779E-08	1.9E-06
A_33_P3254311		TRBV30	-3.93653	1.1874E-09	6.21E-08	A_33_P3587376	**SNAR-A3**	SNAR-A11	2.186417	3.0524E-07	4.66E-06
A_19_P00322254	**MEG3[39,40]**	MEG3	-3.79877	3.2003E-09	1.3E-07	A_33_P3346618		LOC441179	2.086074	6.2215E-10	3.86E-08
A_32_P125832	**LOC100128893**	LOC100128893	-3.7141	3.2143E-07	4.86E-06	A_19_P00316552		LOC100505634	2.067297	1.0161E-10	1.14E-08
A_19_P00321493		LOC100505920	-3.50828	2.0406E-10	1.83E-08	A_19_P00321743		LOC100130938	2.027003	1.6122E-11	3.04E-09
A_33_P3337131	**LOC648691**	LOC648691/IGL	-3.33658	6.638E-10	4.03E-08	A_19_P00320525		LINC00340	2.026397	3.9334E-10	2.78E-08
A_23_P256965	**C8orf12**	C8orf12	-3.29335	2.6132E-09	1.11E-07	A_19_P00322992		LOC100128130/LOC100505634	2.014693	2.3139E-11	3.9E-09
A_33_P3347201		LOC285878	-3.25336	7.0915E-13	3.42E-10	A_33_P3308872	**POM121L1P**	POM121L7	2.001845	2.5039E-10	2.07E-08
A_33_P3356935	**C11orf92**	C11orf92	-3.21639	7.6119E-11	9.4E-09	A_19_P00322351		LINC00340	1.871491	2.8462E-07	4.4E-06
A_19_P00805664		LOC283177	-3.13059	6.3405E-07	8.43E-06	A_24_P367602	**DUSP5P**	DUSP5P/RHOU	1.854605	3.229E-06	3.21E-05
A_19_P00811951		LOC219731	-3.08071	2.3571E-09	1.04E-07	A_19_P00812924		LOC100507165	1.854221	0.00023033	0.001079
A_32_P46594	**LOC145837**	LOC145837	-3.07454	1.1611E-08	3.42E-07	A_33_P3234472	**LOC284751**	LOC284751	1.827521	5.5453E-06	5.01E-05
A_33_P3273233		LOC100129215	-3.03253	7.9106E-10	4.52E-08	A_19_P00319050		LOC100505634	1.8246	5.4948E-10	3.53E-08
A_23_P106069		TRA/TRAV6	-3.02961	2.345E-09	1.03E-07	A_19_P00319503		LOC100505634	1.817392	3.7654E-10	2.72E-08
A_33_P3238182	**C8orf71**	C8orf71	-3.02492	1.0344E-07	1.96E-06	A_24_P892494	**LOC285548**	LOC285548	1.816767	0.00208747	0.006787
A_33_P3218004	**AKR7L[41]**	AKR7L	-3.00994	3.1722E-06	3.17E-05	A_24_P203328	**TPRXL**	TPRXL	1.804866	5.5251E-05	0.000328
A_33_P3299042		GOLGA8DP	-2.95988	2.1028E-11	3.67E-09	A_33_P3312384	**C21orf34**	LINC00478	1.794868	0.00085068	0.003215
A_33_P3330074		TRPC2	-2.90548	8.0275E-09	2.54E-07	A_32_P62963	**LOC400578**	KRT16P2	1.756419	0.00012535	0.00065
A_33_P3214052		LOC729178	-2.82152	3.7874E-11	5.59E-09	A_33_P3371325		FTH1P19	1.750069	1.3487E-05	0.000103
A_19_P00326795		LOC100288428	-2.81487	1.0289E-09	5.55E-08	A_33_P3404316	**LOC399959**	MIR100HG/LOC100507145	1.716606	0.0016418	0.005566

Log2FC<0: down-regulated (left panel), Log2FC>0: up-regulated (right panel)

In our previous report [36], the fold change (FC) of H19 in 74 gastric cancer versus paired noncancerous tissues was 6.015, with a *P*-value of 0.017. This result was consistent with the data of H19 (Absolute FC = 6.06) in this microarray analyses. Furthermore, over-expression of H19 contributes to the proliferation, migration, invasion and metastasis of gastric cancer.

### Gene Ontology categories

**Fig 2:**
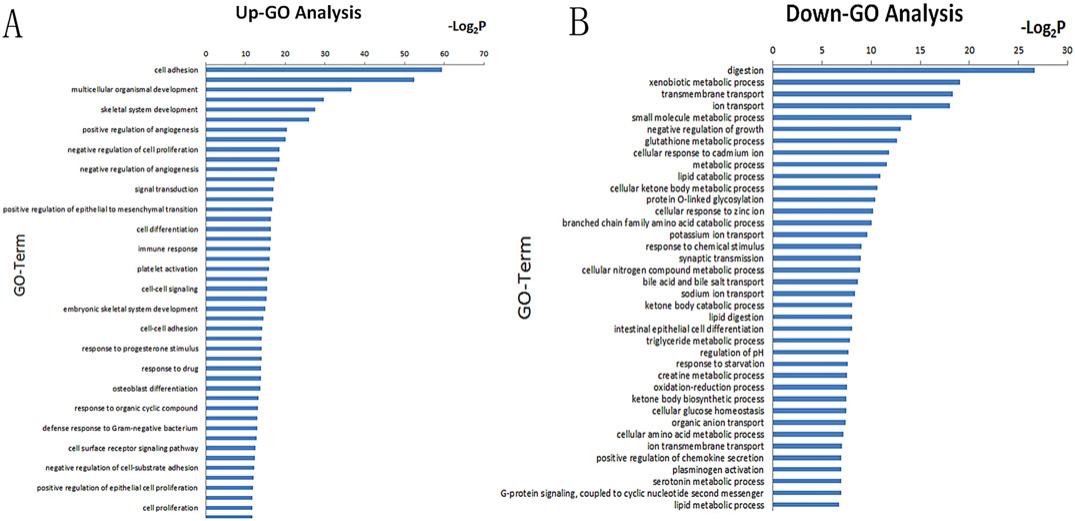
GO categories based on differential genes in the expression microarray. **A)** The significant GO categories for up-regulated genes. **B)** The significant GO categories for down-regulated genes. *P*-value <0.01 and FDR <0.01 were used as a threshold to enroll significant GO categories.

All the differentially expressed genes were classified into different functional categories according to the Gene Ontology (GO) project for biological processes. Based on our microarray data, GO analyses indicated that 208 GO terms were enriched (*P*<0.01, FDR<0.01) (S1 Table). The primary GO categories for 170 up-regulated GO terms were focused on cell adhesion, angiogenesis, multicellular organism development, axon guidance, skeletal system development, collagen fibril organization, positive regulation of angiogenesis, wounding and negative regulation of cell proliferation (Fig 2A). The main GO categories for down-regulated genes were digestion, xenobiotic metabolic process, transmembrane transport, ion transport, small molecule metabolic process, negative regulation of growth, glutathione metabolic process, cellular response to cadmium ion and metabolic process (Fig 2B).

**Fig 3:**
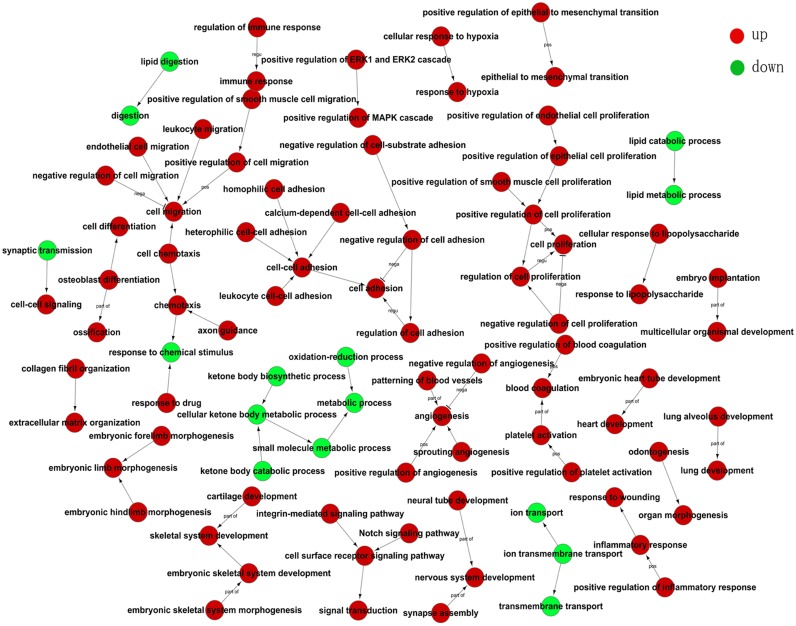
Interaction of GO categories (GO Tree) based on analyses of differential GO categories. Red dots represent up-regulated GO categories and green dots represent down-regulated GO categories.

According to the differential genes and functions, we built a GO Tree to explore the interactions among all the differential GO categories. The diversity in these categories when comparing cancerous and control tissues suggested that gastric cancer may be associated with significantly up-regulated cell migration, cell proliferation, angiogenesis, cell—cell adhesion and cell surface receptor signaling pathways, while cell metabolism processes and ion transmembrane transport are down-regulated (Fig 3).

### Pathway analyses

**Fig 4:**
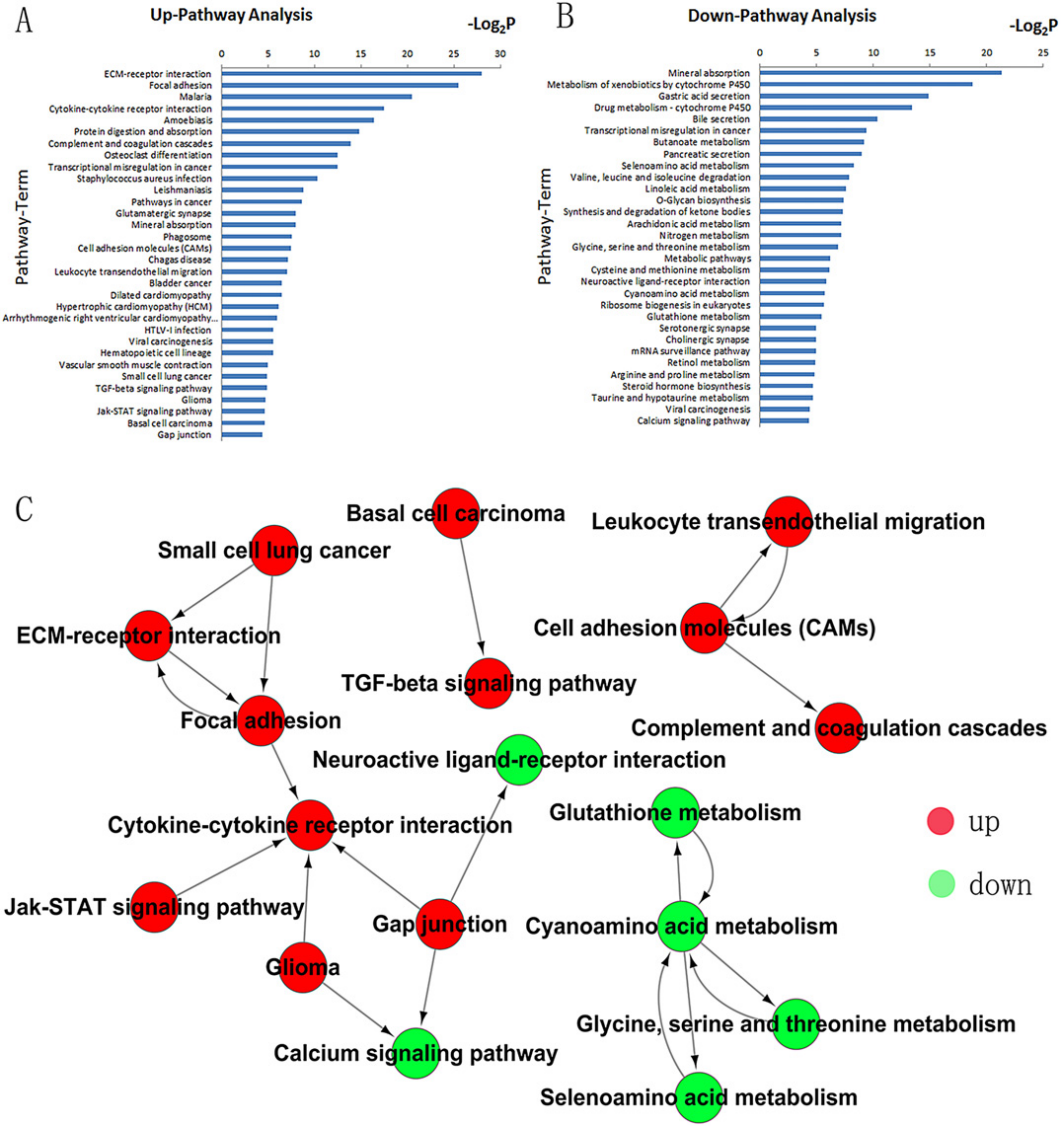
Pathway analysis of differentially expressed genes according to the KEGG database. **A)** Top-ranking up-regulated pathways identified by KEGG. **B)** Top-ranking down-regulated pathways identified by KEGG. Differential pathways are listed according to *P*-value <0.01 and FDR <0.01.**C)** Pathway-Act network showing the interaction of differential pathways. The red dots represent up-regulated pathways and the green dots represent down-regulated pathways.

Pathway analyses were used to identify the significant pathways associated with the differentially expressed genes according to KEGG. There were 32 up-regulated pathways and 31 down-regulated pathways based on our data (Fig 4). Furthermore, the pathway profiling was consistent with the results for the GO categories in cancer-related biological functions. Our data showed some differential genes highly up-regulated which suggested their involved pathways were activiated. For example, SFRP4, WNT11, FZD2, MYC were highly expressed in cancer tissues which represent the Wnt pathway was activiated and BCL2A1, ICM1, TNFSF14 in NF-κB pathway were highly expressed as well. Most of the cancer-related signaling pathways such as JAK/STAT, Wnt, NF-κB, PI3K, mTOR, Hedgehog and Notch pathways were activated in gastric cancer compared with noncancerous tissues based on our data (S2 Table). The up-regulated pathways which were focused on cell adhesion, transcriptional dysregulation, carcinogenesis and differentiation were correlated with tumorogenesis and metastasis (Fig 4A). However, the down-regulated pathways were generally responsible for metabolism (Fig 4B).

### Gene-Act network

**Fig 5:**
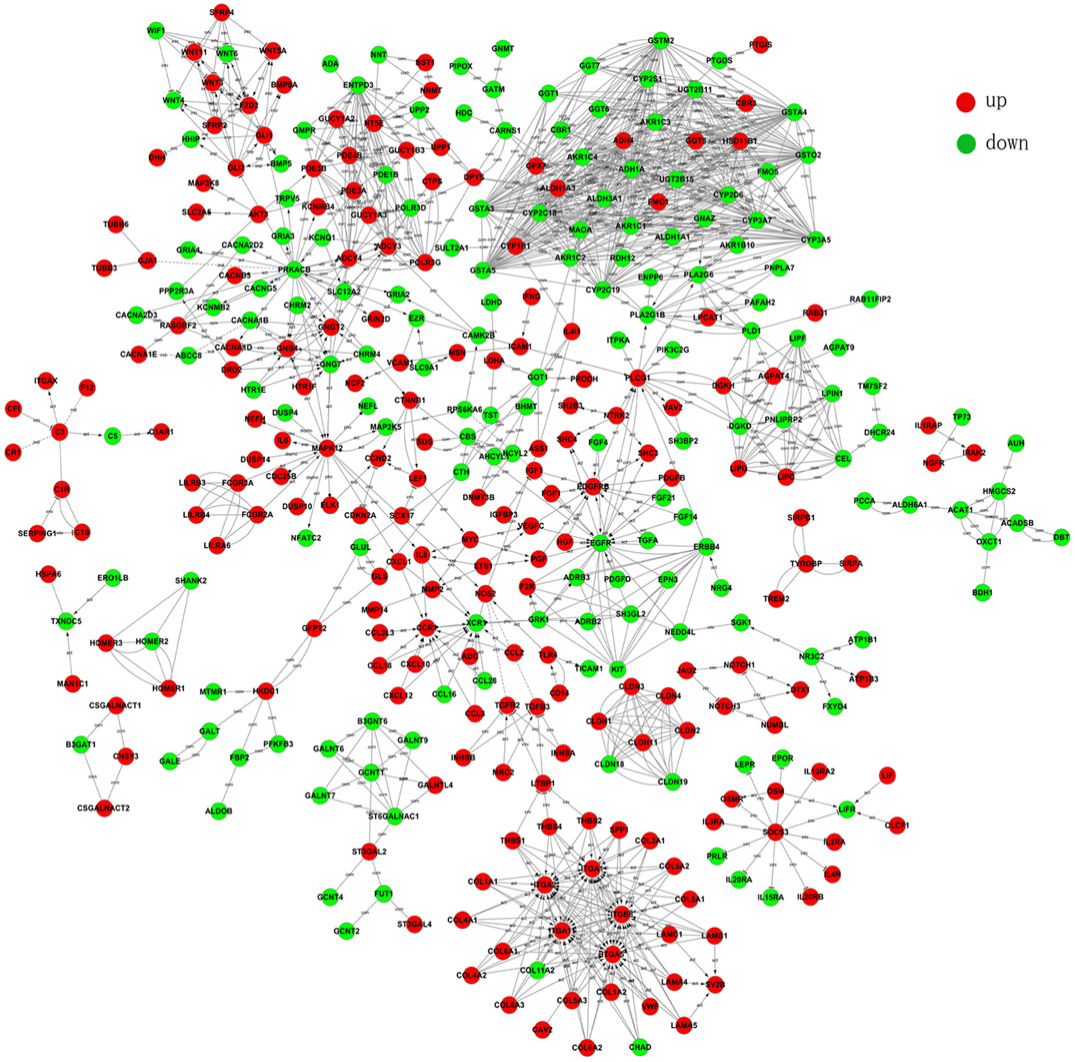
Gene-Act network of differential genes according to pathways in the database. Red dots represent up-regulated genes and green dots represent down-regulated genes. The arrows indicate the connection and regulatory relationship between two genes. Genes that have more connections with other genes have a higher degree score.

Based on GO categories and pathway analysis, one gene may be involved in several pathways or interact with several other genes. We pooled the differential genes and built a network of the interactions of differentially expressed genes. A high degree protein regulates or is regulated by many other proteins, which implies an important role in the Gene-Act network (S3 Table). The glutathione S-transferase (GST) family, cytochrome P450 (CYP) family, UDP glucuronosyltransferase 2 (UGT2) family, Epidermal Growth Factor Receptor (EGFR) family and cAMP-dependent protein kinase catalytic beta (PRKACB) were at the core of the gene—gene interaction network. They may play key roles in the network because they possessed the strongest degree (degree >25) centralities (gene-gene interactions) (Fig 5). It has been reported that GST, EGFR and PRKACB are responsible for signal transduction pathways involved in tumor growth and differentiation in different type of cancers [42,43].

### Gene co-expression network

**Fig 6:**
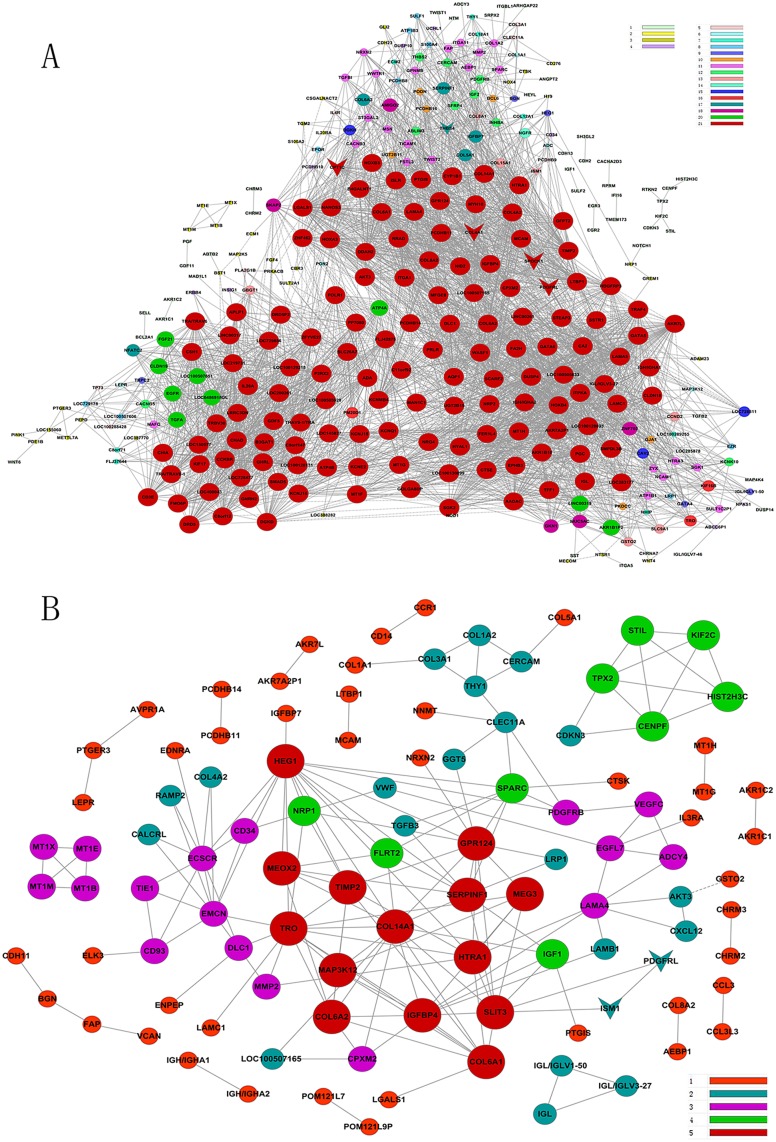
Co-expression network of genes differentially expressed between normal and cancer tissues. **A)** Co-expressed genes and their network in noncancerous tissue. **B)** Co-expressed genes and their network in gastric cancer. The greater the value of K-score, the stronger the differentially expressed genes are co-expressed. The scale of the K-score is from 1 to 21 in normal tissue but from 1 to 5 in cancer tissue.

We produced a gene co-expression network based on the differentially expressed genes, proteins and protein complex in cancer tissues and noncancerous (control) tissues, respectively. Compared with the control, the connections between genes in cancer tissues were less, which suggested that most of the physiological gene—gene interactions and linkages in normal tissues had been broken or lost in the cancer tissues (Fig 6A and 6B). The genes with high degree and k-core which means they possessed most of the interactions with other geneswere known as key genes in the interaction network (Fig 6B) including TRO, GPR124, TIMP2, EMCN, SLIT3, HTRA1, SPARC, LAMA4 and MEOX2 (Table 3). They were responsible for cell signaling, adhesion, angiogenesis, migration, growth and metastasis.

**Table 3:** top 20 differential genes with highest Degree and K-Core in co-expression network

Gene Name	Description	Degree	K-core
**HEG1**	HEG homolog 1 (zebrafish)	15	5
**COL14A1**	collagen, type XIV, alpha 1	14	5
**TRO[44]**	trophinin	14	5
**GPR124[45]**	G protein-coupled receptor 124	12	5
**IGFBP4**	insulin-like growth factor binding protein 4	11	5
**ECSCR**	endothelial cell-specific chemotaxis regulator	10	3
**EMCN[47]**	endomucin	10	3
**SERPINF1**	serpin peptidase inhibitor, clade F (alpha-2 antiplasmin, pigment epithelium derived factor), member 1	10	5
**SLIT3[48]**	slit homolog 3 (Drosophila)	10	5
**COL6A1**	collagen, type VI, alpha 1	9	5
**HTRA1[49]**	HtrA serine peptidase 1	9	5
**TIMP2[46]**	TIMP metallopeptidase inhibitor 2	9	5
**COL6A2**	collagen, type VI, alpha 2	8	5
**SPARC[50,51]**	secreted protein, acidic, cysteine-rich (osteonectin)	8	4
**LAMA4[52,53]**	laminin, alpha 4	7	3
**MAP3K12**	mitogen-activated protein kinase kinase kinase 12	7	5
**MEG3[39,40]**	maternally expressed 3 (non-protein coding)	6	5
**MEOX2[54]**	mesenchyme homeobox 2	6	5
**CENPF**	centromere protein F, 350/400kDa (mitosin)	5	4

### Confirmation of microarray results by qPCR

We performed Quantitative Real-time PCR (qPCR) on 6 up-regulated genes (COL1A, BGN, SPP1, MELK, IGFBP4, SPARC) and 4 down-regulated genes (PGC, SST, MT1X, S100P) to verify our data in gastric cancer tissues (Tumor) and noncancerous tissues (Normal). The expression ratios of these 10 genes (Tumor/Normal) from qPCR are consistent with those from microarray (S4 Table). It suggested the data of differential genes expression from microarray was reliable. What’s more, our team has been worked on some of the differential genes such as PHF10[55], CEACAM6[56], SFRP1[57], SOX11[58], CLDN1[59] to investigate their expression and functions in gastric cancer and the results perfect proved our microarray data.

## Discussion

Microarray gene-expression analyses on gastric cancer have previously been used to predict diagnostic markers [60] and to identify gene expression patterns associated with prognosis [61,62], but it hasn’t been used to reveal molecular interactions among lncRNAs and mRNAs in GC. In this study, we analyzed 26 gastric cancer tissues with paired noncancerous tissues and profiled the genes differentially expressed according to their GO categories, pathways, Gene-Act network and Co-Expression network.

The gene expression results were obtained by using an Agilent G3 Human GE 8x60K microarray, which not only covers the transcriptome databases for mRNA targets but also includes probes for lncRNAs (long non-coding RNAs). With the combination of mRNA and lncRNAs, it can perform two experiments on a single microarray and predict lncRNA function and interaction with mRNAs. The analyses revealed a set of genes that were differentially expressed between gastric cancer and normal tissue. Some of them have been reported previously in gastric or other cancers. For example, expression of gastrokine-2 (GKN2) was significantly down-regulated or absent in gastric cancer cell lines, gastric intestinal metaplasia, and tumor tissues. Over-expression of GKN2 contributed to cell proliferation, migration, and invasion of gastric cancer and arrested the cell cycle at the G1–S transition phase [6]. In contrast, levels of expression of inhibin beta A (INHBA) were significantly higher in cancer tissue than in adjacent normal mucosa, and it is regarded as an independent prognostic factor in gastric cancer [22]. In addition, we discovered some novel genes, such as TMEM184A, PSAPL1, KIAA1199, CLRN3 and FNDC1, which have not been reported in gastric cancer previously, and their roles in cancer remain unknown.

One of the advantages of our gene expression microarray analysis is that it represented the expression of lncRNAs and mRNAs so that both could be investigated together. Our previously report on the role of lncRNA H19 and its network in GC[36] was based on this microarray data. However, most of the lncRNAs such as DRD5, FMO6P, SNAR-A3 and TPRXL showed in our microarray haven’t been identified and need further investigation to clarify their roles in gastric cancer.

Based on our gene expression profiling data, the genes and their functions activated in gastric cancer were responsible for proliferation, adhesion, migration and metastasis, which was consistent with the results from pathway analyses. Interestingly, we discovered that most of the cancer-related signaling pathways reported previously such as Notch, mTOR and Hedgehog were activated in GC based on our data. These results support the viewpoint that heterogeneity is the characteristic of GC. Comparison of the co-expression network between normal tissues and cancer suggested that the expression, functions and interactions of the majority of physiological gene were lost or damaged in gastric cancer, whereas proliferation, migration and metastasis were abnormally enhanced. These interesting findings match the characteristics of cancer, such as anaplasia and dedifferentiation. These differentially expressed genes involved in signaling pathways acted as key genes in co-expression network might be the potential targets of anti-cancer therapy or diagnostic markers in the future.

## Supporting Information

S1 Table Pathway analyses of differential genes (XLSX)

S2 Table GO analyses of differential genes (XLSX)

S3 Table Gene-Act network of differential genes (XLSX)

S4 Table Primers and verification (DOCX)

S5 Table Methods and Materials (DOCX)
