# Supplementary material for: Characterization of Differentially Expressed Genes Involved in Pathways Associated with Gastric Cancer
Source: PLoS One. 2015 Apr 30;10(4):e0125013. doi: 10.1371/journal.pone.0125013 (PMC4415781; doi:10.1371/journal.pone.0125013)
Supplement: S4 Table — (DOCX) [file pone.0125013.s004.docx]

Table4 Primers of 10 genes for qPCR

| Gene ID | Name | Status | Forward | Reverse |
| --- | --- | --- | --- | --- |
| 1277 | COL1A | up | 5'CACCAATCACCTGCGTACAGA3' | 5'CAGATCACGTCATCGCACAAC3' |
| 633 | BGN | up | 5'CGATGGCCTGAAGCTCAACTA3' | 5'TCGATGGCCTGGATTTTGTT3' |
| 6696 | SPP1 | up | 5'ACACATATGATGGCCGAGGTG3' | 5'TGGATGTCAGGTCTGCGAAA3' |
| 9833 | MELK | up |  |  |
| 3487 | IGFBP4 | up | 5'TTCCTTCACTCATCCAGCCAC3' | 5'AGGAGTGTCTCCACATGCCAA3' |
| 6678 | SPARC | up | 5'TGGACTCTGAGCTGACCGAAT3' | 5'GATCTTCTTCACCCGCAGCTT3' |
| 5225 | PGC | down | 5'ACCGGCTTCTTTGGCTATGA3' | 5'GCCATCAAACTGCGCATAGA3' |
| 6750 | SST | down | 5'GAAGCAGGAACTGGCCAAGTA3' | 5'CTGCAGCTCAAGCCTCATTTC3' |
| 4501 | MT1X | down | 5'TCTCCTTGCCTCGAAATGGA3' | 5'TGCACTTGTCTGACGTCCCTT3' |
| 6286 | S100P | down | 5'TGGAAAAGACAAGGATGCCGT3' | 5'TTGCAGCCACGAACACGAT3' |


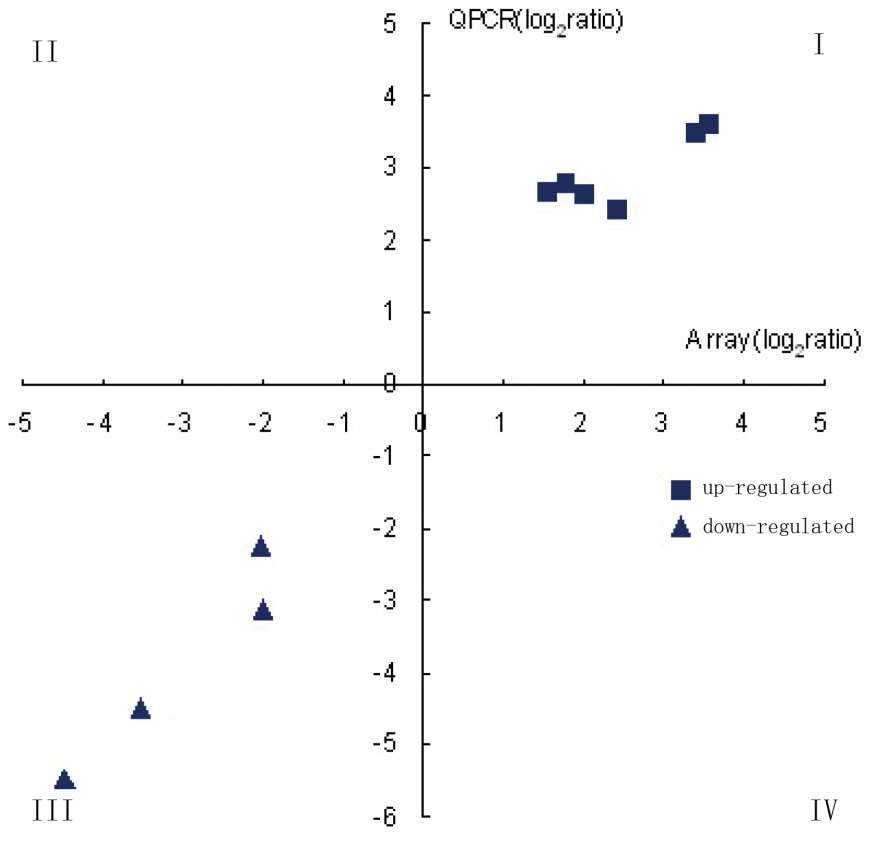


**Figure7.** Verification of the expression of 10 differential genes. The X-axis represented the expression in microarray and Y-axis represented the expression in qPCR. The expression of 6 up-regulated genes was showed as square in I quadrant while the expression of 4 down-regulated genes was showed as triangle in III quadrant.
