# Supplementary material for: Characterization of Differentially Expressed Genes Involved in Pathways Associated with Gastric Cancer
Source: PLoS One. 2015 Apr 30;10(4):e0125013. doi: 10.1371/journal.pone.0125013 (PMC4415781; doi:10.1371/journal.pone.0125013)
Supplement: S5 Table — (DOCX) [file pone.0125013.s005.docx]

**Methods and Materials**

***Microarray experiments***

Total RNA was isolated and amplified using a Low Input Quick Amp Labeling Kit, One-Color (Cat#5190-2305, Agilent technologies, US). Then, the labeled cRNAs were purified by a RNeasy mini kit (Cat#74106, QIAGEN, Germany), followed the instructions of the manufacturer.

Each slide was hybridized with 600ng Cy3-labeled cRNA using a Gene Expression Hybridization Kit (Cat#5188-5242, Agilent technologies, Santa Clara, CA, US) in a Hybridization Oven (Cat#G2545A, Agilent technologies, Santa Clara, CA, US), according to the manufacturer’s instructions. After 17 hours of hybridization, the slides were washed in staining dishes (Cat#121, Thermo Shandon, Waltham, MA, US) with the Gene Expression Wash Buffer Kit (Cat#5188-5327, Agilent technologies, Santa Clara, CA, US), following the manufacturer’s instructions.

Slides were scanned using an Agilent Microarray Scanner (Cat#G2565CA, Agilent technologies, Santa Clara, CA, US) with default settings, Dye channel: Green, Scan resolution=3μm, 20bit, and using Feature Extraction software 10.7 (Agilent technologies, Santa Clara, CA, US). The raw data were normalized using the Quantile algorithm, Gene Spring Software 11.0 (Agilent technologies, Santa Clara, CA, US).

***Limma***

To find differentially expressed genes, we used linear models and empirical Bayes methods to analyze the data. The method is similar to a standard t test for each probe except that the SES are moderated across genes to get more stable results. This prevented a gene with a very small fold change from being judged as differentially expressed just because of an accidentally small residual SD. The resulting *P*-values were adjusted using the BH FDR algorithm.

***GO category***

Gene Ontogeny (GO) analyses was applied to analyze the main functions of the differentially expressed genes using the key functional classification of The National Center for Biotechnology Information (NCBI). Generally, Fisher’s exact test and the *χ*^2^ test were used to classify the GO category, and the false discovery rate (FDR) was calculated to correct the *P*-value; the smaller the FDR, the small the error in judging the *P*-value. The FDR was defined as$FDR=1-\frac{Nk}{T}$, where *N_k_* refers to the number of Fisher’s test *P*-values less than the *χ*^2^ test *P*-values. We computed *P*-values for the GOs of all the differentially expressed genes. Enrichment provides a measure of the significance of the function: as the enrichment increases, the corresponding function is more specific, which helps to find those GOs with a more concrete function description in the experiment. Within the significant category, the enrichment Re was given by: Re=(*n_f_*/*n*)/(*N_f_*/*N*), where *N_f_* is the number of differential genes within the particular category, *n* is the total number of genes within the same category, *N_f_* is the number of differential genes in the entire microarray, and *N* is the total number of genes in the microarray.

***Pathway analyses***

Pathway analysis was used to find the pathways significantly associated with the differentially expressed genes. Pathway annotations of the microarray genes were downloaded from KEGG (http://www.genome.jp/kegg/). A Fisher exact test was used to find the significant enrichment pathways. The resulting *P*-values were adjusted using the BH FDR algorithm. Pathway categories with a FDR <0.05 were reported. Enrichment provides a measure of the significance of the function: as the enrichment increases, the corresponding function is more specific, which helps to find the more significant pathways in the experiment. The enrichment was given by: enrichment=$(\frac{ng}{na})$/$(\frac{Ng}{Na})$ where *n_g_* is the number of differential genes within the particular pathway, *n_a_* is the total number of genes within the same pathway, *N_g_* is the number of differential genes which have at least one pathway annotation, and *N_a_* is the number of genes which have at least one pathway annotation in the entire microarray.

***Gene-Act network***

The KEGG database was used to build the network of genes according to the relationships among the genes, proteins and compounds in the database. The role of each protein in the network was measured by counting its connections to upstream and downstream proteins, known as in-degree (upstream connections) and out-degree (downstream connections), finally obtaining the degree (for all connections) to score each protein. We pooled all the gene–gene interactions together, based on the differential pathways, to reveal the cell signaling and key regulatory genes.

***Co-expression network***

We used gene co-expression networks to find the interactions among genes. Gene co-expression networks were built according to the normalized signal intensity of specific genes. For each pair of genes, we calculated the Pearson correlation coefficient and chose the significantly correlated pairs to construct the network. Within the network analyses, degree centrality is the simplest and most important measure of the centrality of a gene within a network, which determines its relative importance. Degree centrality is defined as the number of links one node has to another. Moreover, to study various properties of the networks, k-cores (from graph theory) were introduced as a method of simplifying the graph topology analyses. A k-core of a network is a sub-network in which all nodes are connected to at least k other genes in the sub-network. A k-core of a protein–protein interaction network usually contains cohesive groups of proteins. The purpose of network structure analyses is to locate core regulatory factors (genes) in one network. Core regulatory factors connect most adjacent genes and have the highest degrees. While considering different networks, core regulatory factors were determined by the differences in degree between two class samples. These always possess the largest differences in degree.
